# Optimization of Care Pathways Through Technological, Clinical, Organizational and Social Innovations: A Qualitative Study

**DOI:** 10.1177/11786329231211096

**Published:** 2023-11-09

**Authors:** Jean-Baptiste Gartner, André Côté

**Affiliations:** 1 Département de management, Faculté des sciences de l’administration, Université Laval, Québec, QC, Canada; 2 Centre de recherche en gestion des services de santé, Université Laval, Québec, QC, Canada; 3 Centre de recherche du CHU de Québec, Université Laval, Québec, QC, Canada; 4 Centre de recherche du CISSS de Chaudière-Appalaches, Québec, QC, Canada; 5 VITAM, Centre de recherche en santé durable, Université Laval, Québec, QC, Canada; 6 Centre de recherche de l’Institut Universitaire de Cardio-Pneumologie de Québec, Université Laval, Québec, QC, Canada

**Keywords:** Care pathways, infusion of innovation, practices transformation, implementation science, scaling up, healthcare management

## Abstract

Numerous calls at national and international level are leading some countries to seek to redesign the provision of healthcare and services. Care pathways have the potential to improve outcomes by providing a mechanism to coordinate care and reduce fragmentation and ultimately costs. However, their implementation still shows variable results, resulting in them being considered as complex interventions in complex systems. By mobilizing an emerging approach combining action research and grounded theory methodology, we conducted a pilot project on care pathways. We used a strongly inductive process, to mobilize comparison and continuous theoretical sampling to produce theories. Forty-two interviews were conducted, and participant observations were made throughout the project, including 60 participant observations at meetings, workshops and field observations. The investigators kept logbooks and recorded field notes. Thematic analysis was used with an inductive approach. The present model explains the factors that positively or negatively influence the implementation of innovations in care pathways. The model represents interactions between facilitating factors, favourable conditions for the emergence of innovation adoption, implementation process enablers and challenges or barriers including those related specifically to the local context. What seems to be totally new is the embodiment of the mobilizing shared objective of active patient-partner participation in decision-making, data collection and analysis and solution building. This allows, in our opinion, to transcend professional perspectives for the benefit of patient-oriented results. Finally, the pilot project has created expectations in terms of spread and scaling. Future research on care pathway implementation should go further in the evaluation of the multifactorial impacts and develop a methodological framework of care pathway implementation, as the only existing proposition seems limited. Furthermore, from a social science perspective, it would be interesting to analyse the modes of social valuation of the different actors to understand what allows the transformation of collective action.

## Background

Many calls have been made over the past 20 years to improve the organization and quality of care in countries such as Canada^1^ and the United States,^2-4^ as well as internationally by the World Health Organization.^5,6^ However, suboptimal performance and inadequate use of resources persist.^7-9^ These problems seem to stem largely from persistent organizational, professional and data silos,^10^ leading to disruptions in the continuity of health services,^11^ unnecessary waiting times,^12,13^ defects in the flow of information between episodes,^14^ and the performance of examinations that may be unnecessary.^15^ They come also from difficulties in innovating and integrating evidenced-based knowledge into routine clinical practice.^16^ Thus, the need to improve the quality of care has led to interest in initiatives to redesign the provision of care and give greater social value.^17^ Indeed, modern systems that can integrate innovation and knowledge in daily care have the potential to better contain costs and improve population health.^18^

Since the emergence of the concept, care pathways have been intended to improve outcomes by providing a mechanism to coordinate care and reduce fragmentation and ultimately costs.^19^ Many efforts have already been developed to better define and characterize the concept.^20-23^ Although encouraging results have been shown recently,^12^ the results of care pathway optimization projects remain variable and sometimes disappointing in relation to the resources invested.^8^ This variability in results leads experts to view these programmes as complex interventions in complex systems.^23^ This is why it seemed appropriate to redefine and characterize the concept. This new definition, simplified here, considers the care pathway as ‘a long-term and complex managerial intervention adopting a systemic approach, for a well-defined group of patients who journey across the entire continuum of care, from prevention and screening to recovery or palliative care’.^22^

From there, it becomes necessary to test this approach in a pilot project to verify the relevance of such a framework that considers operational, organizational and social realities. In addition, most studies are still designed and implemented to respond to local realities, highlighting possible weaknesses in spreading.^24,25^

Based on an in-depth analysis of a care pathway optimization pilot study in Quebec, the aim of this article is to develop a model for the optimization of care pathways which integrates favourable factors, challenges and barriers to implementation.

## Methods

We opted for an emergent study design in healthcare combining action research and grounded theory methodology.^26,27^ An action research project^28^ was carried out collaboratively between a specialized healthcare organization and the research team. As further described in the action research project section, a group of multidisciplinary researchers and stakeholders of the organization managed a care pathway implementation project and researchers explored and analysed the process guided by grounded theory methodology.^29-32^

### Setting

The setting is an ultra-specialized hospital centre in the province of Quebec, Canada. Committees were created comprising both organizational and research group stakeholders but also a representative of the decision makers, a patient partner and a representative of the private partner. In total, nearly a 100 people participated directly in the project including nineteen university professors, supported by some 30 research professionals and students, in various faculties and disciplines. On the organizational side, managers from all levels, clinicians from all the professions involved and some support professionals were involved. Finally, the private partner, able to support the deployment of the technology was present throughout the project. In this document, the term ‘staff’ refers to all categories of professionals. Where reference is made to a particular category of staff, this is specified.

### Ethical process and consent to participate

The ethical review committee of the organization gave a favourable review for the original study on 23 June 2022 [Reference no. 2022-3826, 22200]. Several measures were implemented, including obtaining written consent from participants, the method of storing data, commitments to confidentiality and anonymity of participants. Participation in this study was voluntary.

### The action research project

Based on the initiative of a patient partner, a pilot project was launched in early 2022. The objective was to optimize the care pathways by reducing the time allocated to clinical practices through a significant transformation in the adoption of technological, clinical, organizational and social innovations. Technological innovations consisted of: (i) formalizing and standardizing clinical care; (ii) minimizing administrative and clerical tasks; and (iii) making the required information available and accessible. Clinical innovations consisted of: (i) putting in place tools to facilitate fluid interactions between clinical actors; and (ii) using the best clinical practices based on evidence. Organizational innovations aimed to implement modes of coordination and communication to ensure a better flow of activities. Social innovations aimed to promote a culture that emphasizes: (i) shared responsibility; (ii) evidence-based knowledge generation; and (iii) continuous learning. Three care pathways for pulmonary pathologies were targeted based on volume of activity. The care pathways for chronic obstructive pulmonary disease, pulmonary fibrosis and pneumonia were the focus of the optimization project.

The project was carried out in 3 main phases, each of which included cycles of ‘observation’, ‘reflection’, ‘planning’ and ‘action’, which are significant for action research.^28^ In line with the learning health system approach and its learning cycle,^33^ the first phase consisted of the design and initiation which mobilized the development of the project and the study of the activity to define the target population (data collection). The second phase consisted of the analysis of the current organization and patient journey and the synthesis of knowledge contained in international clinical practice guidelines (creation of knowledge). The third phase consisted of the co-construction of solutions and their implementation in the environment (knowledge mobilization).

### Participants and data collection

In line with the grounded theory approach,^30-32^ we used a strongly inductive process, to mobilize comparison and continuous theoretical sampling in order to produce theories.^32^ Forty-two interviews were conducted throughout the project, 18 with actors involved in the steering of the project and 24 with voluntary staff to understand the enabling factors and barriers to implementation in each phase. The semi-structured interviews were conducted by the first author using interview guides and lasted between 45 minutes and 1 hour and 15 minutes. To ensure anonymity, an interview code was given, from 01 to 42 followed by 2 letters for the stakeholder’s role. Participant observations were made by the 2 authors throughout the project, including, but not limited to, 60 participant observations at meetings, workshops and field observations representing over 150 hours of observation. The investigators kept logbooks and recorded field notes. These 2 materials included activity calendars and reflections.^28^ Additional File 1 provides questions included in the different semi-structured interview guides. See Table 1 for a complete overview of data collection.

**Table 1.** Overview of the data collection in the project.

Data types	Description of the content and source of the data	Sampling details
Semi-structured interviews	Forty-two semi-structured interviews were conducted throughout the project. Eighteen of these interviews were with actors involved in the governance of the project. The remaining twenty-four interviews were conducted with professionals and managers in the field. The interviews were all recorded and transcribed before being uploaded to Nvivo (version 12).	• Steering of the project - Six researchers - Five managers - Two patient partners - Two physicians - Two representatives of the decision-makers - A representative of the private partner • Field professionals - Eleven nurses - Six operational managers - Two physicians - One care advisor - One occupational therapist, - One physiotherapist - One Nutritionist - One respiratory therapist
Participatory observations	The authors were directly involved in the whole project and all meetings. Thus, participant observation includes, but is not limited to sixty meetings, several workshops and field observations, representing over 400 h. Field observations were patient shadowing and observation and questions to professionals during their work. The field notes and logbook have been uploaded to Nvivo (version 12).	• Logbook and field notes - The logbook contains summaries and methodological and analytical notes of over 60 meetings and workshops. - The field notes include operating procedures descriptions, analytical notes and reflective notes on observation afterwards.

### Analysis

During the entire action research, a constant comparative analysis was performed.^29^ The concepts emerged inductively from interviews and field notes. The coding and analysis process followed the 6 successive stages proposed by Paillé^34^: coding, categorization, linking, integration, modelling and theorization. Data were processed and analysed independently by the 2 authors using Nvivo (version 12) software. Indeed, this increased the capacity to process very large volumes of data that would have been very difficult to do manually.^35^ The results were then shared and compared for discussion. The preliminary theoretical schema and related narratives were successively reviewed by the 2 authors for internal consistency.

### Rigour

Several approaches were mobilized for rigour. Triangulation was achieved mobilizing as many different methodological perspectives as possible.^32^ Validity criteria of Gaudet and Robert were used, which follow 3 successive phases: vertical analysis, horizontal and theorizing analysis.^31^ In addition, particular attention was given to the 3 criteria of Denzin’s causal proposition: the temporal order between variables, covariance and the exclusion of rival causal factors.^32^ The researcher’s reflexivity was supported by the use of the logbook and field notes.^36^ Finally, theoretical sampling was evaluated and questioned throughout the research process.^30^

## Results

**Figure 1.**
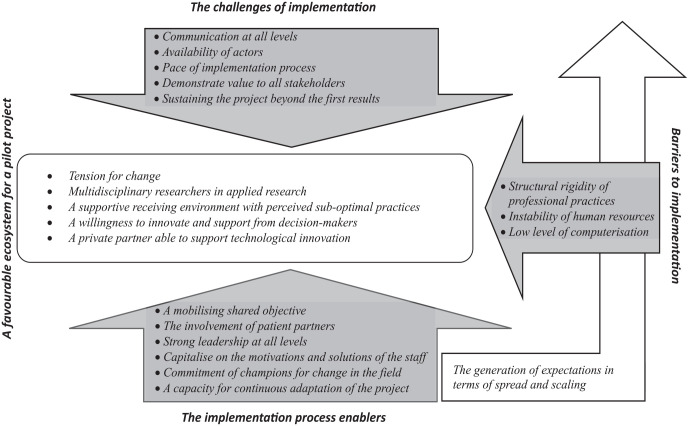
Conceptual model of the enablers, challenges and barriers to implementing innovations in care pathways.

The in-depth analysis of this case study has enabled us to develop a detailed understanding of enabling factors, challenges and barriers to the implementation of care pathways. Thus, a first model for the implementation of care pathway optimization is emerging (see Figure 1). As indicated in each title, all categories of the conceptual model include several subcategories. The detailed results are based on the different data sources. The narrative includes some quotes from participants. Additional File 2 provides further examples of quotes, as well as the types of actors who raised the concept.

### A favourable ecosystem for a pilot project

Initiated by a desire on the part of a patient partner, his motivation met several favourable cumulative factors that helped to create a favourable ecosystem for the emergence of the pilot project. This ecosystem represents prerequisites that need to be analysed during the design phase, and on which few actions can modify the characteristics during the analysis and implementation phases.

#### Tension for change

Exacerbated by the COVID 19 pandemic, healthcare organizations in Quebec are subject to significant pressure on human resources, due to a lack of available human resources, a heavy administrative burden and recently important changes linked to adaptation to the pandemic context, findings widely shared by the staff. Thus, this project arrived in a favourable context of tension for change, as summarized by this strategic manager during a meeting: *‘*5 *years ago, if we had done this project, it would have been very difficult. Today, we are elsewhere, we have a very good momentum to do it’*. This element was influential, both in the birth of the project and in the motivation of the staff to participate actively.

#### Multidisciplinary researchers in applied research

Although the research has long aimed to be independent of the object of the study, more and more researchers are keen to do applied research. In our project, we were able to capitalize on researchers in multiple disciplines, wishing to have a tangible impact, as one of the principal investigators explains: *‘I’m not interested in doing research in a vacuum, anymore, it’s useless. I want to do research in care environments where knowledge is useful for solving problems for patients and professionals’* (25PI). Thus, the research team was composed of researchers from a wide range of disciplines with a shared desire to have a beneficial impact on care delivery.

#### A supportive receiving environment with perceived suboptimal practices

Furthermore, it was equally essential to have a receiving environment that is fully convinced of the relevance of the approach and ready to support it fully, as one strategic manager confirmed: *‘I am delighted with this project, so it is becoming one of my priorities. And you can always count on me to unblock the deadlocks’* (32SM). Nevertheless, staff awareness of the existence of suboptimal practices was a factor that contributed to buy in and commitment during the implementation phase (see Additional File 2 for the detailed information on perceived suboptimal practices).

#### A willingness to innovate and support from decision-makers

The presence of the necessary resources was vital for the conduct of the project. Therefore, decision makers needed to be convinced of the innovative nature of the approach, as stated by a representative of the decision makers: ‘*Bring this dimension of care pathways and innovation into the care pathway. It was a promising project because it brought together several expertise and an IT company was involved’* (27DM). This support has been essential to ensure the presence of material and financial resources over time.

#### A private partner able to support technological innovation

Finally, it was necessary to rely on a private partner with the skills and infrastructure to deploy current technological solutions that required the use of software and applications that allowed task automation.

### The implementation process enablers

Several key elements facilitated the implementation of the project throughout its rollout, from design, to analysis, to implementation. These are factors which the project stakeholders can influence to ensure the success of the pilot project.

#### A mobilizing shared objective

Although the objectives of the project were numerous, staff and managers needed to focus on a shared objective capable of transcending professional perspectives to avoid falling into power struggles between professional bodies, as supported by a strategic manager during a meeting: *‘It’s not just a change for the nurses, for the doctor, for the occupational therapist, whatever, but it’s a change for the patient. And that’s what we had to put forward all the time’.* Thus, the patient as a primary concern, emerged as a unifying objective throughout the project.

#### The involvement of patient partners

One of the major challenges of the analysis phase was to move away from the siloed perspective carried by each of the professional actors. To enable this, a patient partner was involved in data analysis and field observations, as this researcher points out: ‘*We have reached a stage where we need to consult not only health professionals, but also patients, because their vision is different from ours’* (35RS). His participation in committees and workshops allowed him to systematically question the appropriateness of a decision or organization for the delivery of patient care.

#### Strong leadership at all levels

Within both the research team and the receiving environment, the leadership of the main actors has been put to the test, as one strategic manager explains: *‘It takes strong leadership, a size that ensures the follow-up, objectives, the deadlines and therefore leadership at all levels. It’s a long-term project’* (37SM). The duration of the project is a factor that can quickly impact on the commitment of all actors. In fact, both the principal investigators and the strategic managers had to maintain a presence and leadership in the many working meetings to ensure a good level of commitment from all the stakeholders throughout the pilot project.

#### Capitalize on the motivations and solutions of the staff

The use of semi-structured interviews and patient shadowing enabled the staff to express themselves. They expressed their frustrations and motivation to find solutions to give time back to care, as expressed by this assistant head nurse during a field observation: *‘Obviously, the lack of time and I think it creates frustrations as well when there’s not enough time to give quality care to patients’.* This speaking time (in interviews or co-construction workshops) encouraged them to participate and propose solutions, as this doctor points out: *‘Because when they approve it, they participate in it. They buy it. So, they are positive’* (24MD). Indeed, giving the actors a voice and choices was an important lever for commitment.

#### The commitment of champions for change in the field

The choice of workshop participants rested on the shoulders of the receiving environment. They must be able to target the champions for change to improve staff buy in, as this strategic manager indicated: *‘You have to target people, you know, to have change agents and then, often we call them champions, that will contaminate in a positive leader way’* (11SM). We sometimes had to retarget resource persons to improve staff buy-in.

#### A capacity for continuous adaptation of the project

The choice of solutions to be implemented was constantly adapted to the operational, structural and social realities of the receiving environment, as this researcher points out: *‘ We had to be able to readjust and if we saw that it was not working, we should have another approach’* (35RS). Indeed, any sign of rigidity in the organization and application of the solution generated a reaction. Thus, we had to constantly adapt our methodology and the innovation proposals.

### The challenges of implementation

Even though many favourable conditions were present, the project faced several challenges. The challenges of implementation are factors which have an impact on the project, but which can be acted upon to promote the success of the pilot project. These challenges have been a source of negotiation or discussion throughout the project.

#### Communication at all levels

Communication was an issue at several levels. On the one hand, the size of the research team required a great deal of communication to maintain collaboration throughout the project, as one researcher pointed out: *‘I think it’s*, first *of all, the collaboration between the research team, then communication, it’s collaboration, communication for the exchange of information’* (31RS). On the other hand, communication with the receiving environment had to be fluid and transparent at all levels, which was not always obvious, as this strategic manager points out: *‘Involve the teams, involve them in all phases of the project, even if they do not have a concrete action at a phase. But keep them informed of the project’s progress’* (34SM). Finally, communication must also be directed towards the trade unions, who must be informed of the objectives and progress to be able to answer the questions of staff, as one operational manager pointed out: *‘The trade unions can be allies but they can also be people who will also put us off, the union has to be well informed about what we’re doing’* (01OM). In our case, this was not adequately considered.

#### Availability of actors

The availability of the actors was a challenge both for the staff and the stakeholders in the project. The workload of the staff was an issue, as one care professional points out: *‘We all lack the time to do our clinical work, so it’s to be free for projects, it’s difficult’* (20CP). Likewise, uncertainty about the timing of the researchers’ intervention necessarily leads to problems of researcher availability, as raised by a funder’s representative: *‘There are many researchers on the project. The availability of a researcher, that’s an issue’* (36FR). This was a random occurrence, with each researcher having other research projects and personal commitments impacting on their daily scheduling.

#### Pace of implementation process

Once the co-constructed solution had been validated and tested, its implementation in the flow of activity had to be rapid and fluid because the staff had little time available to appropriate the new mechanisms, as this strategic manager notes: *‘When we get to deployment, it has to be fairly fluid, it has to go quickly, otherwise people will drop out’* (37SM). However, the pace of implementation was also an issue for the private partner, with many unforeseen events leading to additional delays, such as the lead times for ordering the technological infrastructure needed to implement technological optimizations.

#### Demonstrate value to all stakeholders

Although a mobilizing objective had been maintained, it was necessary to demonstrate the value created, as soon as possible, as this researcher supports: *‘We have academic researchers, we have a health system, so we have decision makers, managers, health professionals, and we also have a private company. So, I think that everything has to remain a win-win situation for everyone’* (30RS). In our project, the presence of stakeholders with different issues was a source of much discussion. The demonstration of value created must be obvious to all stakeholders, which is not always easy.

#### Sustaining the project beyond the first results

In research projects, the end of the project often corresponds to the achievement of the first results, the end of funding or the production of the first publications. However, the receiving environment still needs to be supported in the use of organizational and technological solutions, as one strategic manager pointed out: *‘Measure it and then maintain it. Because that’s fine for the next* 2 *months. But after that. Sometimes it slips a bit into their old habits and it’s going to take support when we get there’* (34SM).

### Barriers to implementation

Despite significant progress, the pilot project faced several barriers to the implementation of optimizations. In this project, we have defined barriers to implementation as present factors for which few actions are likely to profoundly change the reality during the pilot project. These barriers were as much structural as organizational and social.

#### Structural rigidity of professional practices

The development of interdisciplinarity was an objective of the project. However, discussions about the potential sharing of tasks or shifting of activities from one profession to another was a source of tension, as indicated by this strategic manager: *‘Tensions between the medical team, the nursing team, and the professional team, in the resources of the different direction. Because you might argue about techniques. What defines a professional or medical discipline is its reserved acts’* (26SM). These tensions are formalized in the organizational structure, with a separation of management, and therefore of resources, with any change requiring negotiation.

#### Instability of human resources

The lack of human resources is not only true in the health sector. Thus, our project faced human resource instability, resulting in numerous adjustments and loss of skills, as anticipated by a representative of the decision makers during a meeting: ‘*An issue will be human resources and continuity for the project. So having good expert people who will continue to follow the project throughout’*. This proved to be true with an impressive turnover of research professionals and students but also significant changes in the strategic management positions of the receiving environment.

#### Low level of computerization

Unsurprisingly, the technological infrastructure was not at the desirable level, as indicated by this principal investigator: ‘*I’ve been working in health data for more than* 20 *years. I know what it’s like. It’s not pretty and it hasn’t really improved much’* (25PI). This limited both the data analysis and the implementation of the technological solution.

### The generation of expectations in terms of spread and scaling

Although the pilot project has not yet been replicated and scaled up, many expectations have already emerged. Thus, the potential spread and scaling was desired and foreseen by the decision makers from the beginning of the pilot project, as explained by this representative of the decision makers: *‘We ensure a response to the project, that we can find results that are going to be beneficial to the ministry as well so that we can transport them into other care pathways’* (36RF). The methodological skills developed during implementation are also rare key skills that need to be retained to ensure spread, as this a representative of the decision makers asserts during a meeting: *‘There is also the whole methodological development aspect, and I will tell you, training as well, because we need to train people for this approach to work’.* Indeed, the learnings achieved during the pilot project were numerous and rich, linked to a series of trials and errors, which allowed the adaptation of the innovations to the operational and organizational realities of the receiving environment. These learnings will subsequently allow a more rapid implementation, as a representative of the private partner pointed out: ‘*Then let’s make sure our foundation is solid, taking the time to readjust, to learn from what we’ve done, then from our mistakes, so that the next time we deploy, our solution will be better’* (28PP). Given the resources invested, the greatest fear lies in the fact that the knowledge is not disseminated in scaling, as this representative of the decision makers pointed out: *‘Then afterwards, the scaling up, the learning, what do you do with the knowledge* that is *built? I think that’s the key for many projects, is making sure that in the end it’s not just a document’* (27DM).

## Discussion

This article presents an implementation pilot project of care pathways in a specialized healthcare organization in the province of Quebec, Canada. The generated conceptual model illuminates the new approach of care pathways as complex managerial interventions^22^ that were tested and analysed in this pilot project with a focus on the enablers, challenges and barriers. As this process, to our knowledge, has never been explained before for this approach, the results provide a new understanding that may be useful in future implementation processes.

The conceptual model was generated from empirical findings but resonates well with existing implementation frameworks. For example, some points are in line with the CFIR framework proposed and improved by Damschroder et al^37-39^ In particular, it strengthens the recent adjustments, emphasizing the need to pay attention to the needs of the actors receiving the innovations and to ensure their commitment.^39^ However, certain key points of our model stand out. Firstly, the conceptual framework is specific to healthcare research in the context of care pathway interventions and stands out for the role and place of patients as partners in data collection and analysis, then in the prioritization of solutions and the co-construction of solutions and innovations. Secondly, it stands out for the importance of involving multidisciplinary researchers in applied health. Finally, it stands out for the involvement of stakeholders in the field in formulating proposals and co-constructing innovative solutions. Similarly, our model joins several works in spread and scaling science, in demonstrating a need to consider the social approach,^24^ but also the importance of anticipating scaling, from the design phase of the pilot project.^40^

Considered as a complex intervention,^23^ a care pathway is indeed a long-term managerial intervention mobilizing many multidisciplinary skills and resources. However, this pilot project has identified and characterized favourable conditions for the emergence of innovation adoption. These conditions should not be neglected, as the absence of one of them can be problematic. Consistent with other studies, the presence of a supportive receiving environment with an awareness of suboptimal practices^39^ is crucial for the adherence of staff and the adoption of the innovations. Similarly, the implementation team should not only be multidisciplinary,^41^ but also contain key implementation science skills. Through the implementation process, there must be a mobilizing shared objective, capable of transcending professional perspectives. This social approach to transformation is too often forgotten in technical approaches.^41^ This is why it is essential to focus on the actors, capitalizing on their motivation and willingness to act and mobilizing research methods of participation and co-construction such as interviews, observation and co-construction workshops. Ensuring the involvement in decisions is often a requirement for success.^37,42^ Similarly, the receiving environment must be able to mobilize its champions at all levels with strong leadership, enabling the generation of collective leadership.^43^ These 2 favourable factors are widely shared in the literature.^39,41^

However, implementation is far from being a smooth ride. Many issues were a source of constant negotiation and adaptation. Indeed, over the nearly 2 years of the project, it was difficult to maintain a constant and effective level of communication, which is recommended,^39^ but not always easy. Similarly, the availability of the various actors, depending on expected and unexpected constraints and the pace of the project seemed to be a challenge. Finally, demonstrating value to all stakeholders is not straightforward, as each stakeholder has their own ways of valuing. This point remains underdeveloped in the literature. The present findings also further illustrate that inadequate computerization is a barrier to pathway implementation,^39,44^ as found in other empirical studies.^45^ Finally, although the involvement of patients as consumers of care is common in implementation science, the involvement of patients as partners, both in the collection of data and in its analysis and decision-making throughout the project, seems to be novel in the implementation science literature. This brings a new vision of care pathways and helps to avoid the pitfall of an approach based only on the professionals’ perspective, which is intrinsically incomplete. They also serve as a constant reminder that changes are made primarily to improve patient experience and outcomes. They embody and strengthen the mobilizing shared objective and ensure that the validations and solutions adopted are in the right direction.

With a view to future spread and scaling, the various points raised in our study do not contradict the literature; thus it seems essential to anticipate spread and scaling up,^40^ both on the part of the decision-makers and the research team, insofar as methodological skills and knowledge must be shared^46,47^ and disseminated in order to allow for scaling up.^47^

The limitations of our findings lie in the fact that they are based on a single case study and only consider the perspective of the willing participants. Thus, it is possible that the staff who were most resistant to the optimization project and less likely to participate, may have had a more negative perception of the change. Finally, this study focussed on the process, so, as is frequently the case at this evaluative stage (process evaluation) of an implementation project,^27,44^ the impact on health outcomes and patient experience (outcome evaluation) were beyond the present scope.

## Conclusions

The present model explains in a new way, the factors that positively or negatively influence the implementation of innovations in care pathways. The model represents interactions between facilitating factors, favourable conditions for the emergence of innovation adoption, implementation process enablers and challenges or barriers including those related specifically to the local context. What seems to be totally new is the embodiment of the mobilizing shared objective of active patient-partner participation in decision-making, data analysis and solution building. This allows, in our opinion, to transcend professional perspectives for the benefit of patient-oriented results. However, this needs to be further analysed. Finally, the pilot project has created expectations in terms of spread and scaling, which are relevant premises in the event of scaling up.

Future research on innovation implementation in care pathways should go further in the evaluation of the multifactorial impacts of the redesign and the enabling and disabling factors for spread and scaling up. In addition, it would be interesting to develop a methodological framework of care pathway implementation as the only existing proposition^41^ seems limited by not integrating the social perspective. Furthermore, from a social science perspective, it would be interesting to analyse the modes of social valuation of different actors to understand what allows the transformation of collective action.

## Supplemental Material

sj-docx-1-his-10.1177_11786329231211096 – Supplemental material for Optimization of Care Pathways Through Technological, Clinical, Organizational and Social Innovations: A Qualitative Study Click here for additional data file. Supplemental material, sj-docx-1-his-10.1177_11786329231211096 for Optimization of Care Pathways Through Technological, Clinical, Organizational and Social Innovations: A Qualitative Study by Jean-Baptiste Gartner and André Côté in Health Services Insights

sj-docx-2-his-10.1177_11786329231211096 – Supplemental material for Optimization of Care Pathways Through Technological, Clinical, Organizational and Social Innovations: A Qualitative Study Click here for additional data file. Supplemental material, sj-docx-2-his-10.1177_11786329231211096 for Optimization of Care Pathways Through Technological, Clinical, Organizational and Social Innovations: A Qualitative Study by Jean-Baptiste Gartner and André Côté in Health Services Insights
