# Supplementary material for: Optimization of Care Pathways Through Technological, Clinical, Organizational and Social Innovations: A Qualitative Study
Source: Health Serv Insights. 2023 Nov 9;16:11786329231211096. doi: 10.1177/11786329231211096 (PMC10637140; doi:10.1177/11786329231211096)
Supplement: sj-docx-1-his-10.1177_11786329231211096 – Supplemental material for Optimization of Care Pathways Through Technological, Clinical, Organizational and Social Innovations: A Qualitative Study [file sj-docx-1-his-10.1177_11786329231211096.docx]

**Additional file 1 -** List of topics/questions in the individual interviews

The topics/questions were used as a reminder for the interviewer and not rigorously followed. Based on how the conversations emerged, the questions were adjusted.

| **Topics** | **Types of questions in the different semi-structured interview guides** |
| --- | --- |
| **Motivations for participating in the project** | - How do you position yourself in the project? - What motivated your involvement? - What impact do you expect this project to have on your practices (individual needs and expectations)? |
| **Objectives, needs and expectations of the Optimising Care Pathways project** | - How would you define the Optimising CP project? - What do you think are the objectives of this project? - What could be the benefits of the project? And for which categories of actors (practical)? - Compared to previous projects, which aspects were not fully satisfactory? |
| **Key success factors and barriers** | - What were the factors that enabled the project to progress? - What were the challenges to the success of the project? - What aspects were targeted for improvement in the CP? - What were the barriers to these improvements? - What tensions to this project have arisen? At what level and with which actors? - What means could be used to circumvent or overcome these tensions? What are the key success factors? |
| **Replication and scaling up perspective** | - What do you think are the key points that will enable replication and scaling up? The barriers? - At the end of the project, what criteria will you use to judge the project a success? A failure? |

Questions translated from French. Abbreviations: CP= Care Pathway
