# Supplementary material for: Optimization of Care Pathways Through Technological, Clinical, Organizational and Social Innovations: A Qualitative Study
Source: Health Serv Insights. 2023 Nov 9;16:11786329231211096. doi: 10.1177/11786329231211096 (PMC10637140; doi:10.1177/11786329231211096)
Supplement: sj-docx-2-his-10.1177_11786329231211096 – Supplemental material for Optimization of Care Pathways Through Technological, Clinical, Organizational and Social Innovations: A Qualitative Study [file sj-docx-2-his-10.1177_11786329231211096.docx]

**Additional file 2 -** Examples of quotes from participants and types of actors who raised the concept

Some examples of quotes that indicate the categories, and included subcategories, in the conceptual model.

| **Categories**  (included subcategories) | **Examples of quotes** |  |
| --- | --- | --- |
| **A favourable ecosystem for a pilot project** | |  |
| Tension for change | - Last year, seeing beds available with no resources in front of them to care for these people. We had an emergency room problem, we were in overcapacity, another problem we had was that getting them into the rooms was impossible because we didn't have the staff. (a patient partner, 23PA)  - You should see the phenomenal amount of paperwork, forms, etc. that has to be filled out. That's what it's come to, that's what everyone is wasting a lot of time. (a physician, 24MD)  - But with Covid, there have been a lot of transformations, of practices, we had no choice, we've been at it for two years. It's a language that they hear, we've forced them to change their practices. (an operational manager, 29OM) | Strategic and operational managers  Staff  Researchers  Patient partners |
| Multidisciplinary researchers in implementation science | - And I like the idea of seeing people like you who are in a field completely outside what we are used to seeing, but who can shed new light and make us think about issues that we don't think about because we don't have the same skills, the same training. (a physician, 24MD)  - It was a promising project because it brought together several expertise, several teams. (a representative of the decision-makers, 27DM) | Researchers  Staff  Decision-makers |
| A supportive receiving environment with perceived sub-optimal practices | - There is one thing to be expected in your project, and that is that the deputy director general, is in the project. She's the second in the hospital's organisation chart. She's a person who can say if we have this as an objective, I want us to mobilise to achieve this objective. So, it's sure to help. (a physician, 24MD)  - We don't have the time. We listen to the patient half-heartedly, almost, because we don't have the time to listen to them fully, to meet their needs. (assistant head nurse, 05AN)  - A lot of dubbing a little bit of information I would say written down and we have to transcribe it into computer. You know, there's a lot of dubbing of information that sometimes I think we waste time doing that. (a caregiver, 17CG)  - I saw a horror story recently, of someone falling through the cracks of the system. Every time you say it's not that bad. Maybe, we can explain it. But when you put the episode together. Now you're saying it's totally unacceptable. It doesn't make sense. You know, there were too many holes everywhere. (a physician, 02MD)  - We know that the problems in the care pathways are precisely the transition in care. So, if we don't have this systemic vision that care transits between several, several actors, we won't get anywhere. (a researcher, 25RS)  - You can't always make solutions that are not interoperable. You know, we've always worked in isolation, and we've always done projects that didn't have a global vision. (a patient partner, 23PA)  - You know, they also have a feeling of incompetence in their work, and they don't have a feeling of accomplishment because they don't do all the tasks that they should be doing and that their expertise allows them to do, because we also have notes that are still handwritten; software in which we have to decide things with more patients, so there is a lot of bureaucracy. (a representative of the funder's representative, 36FR) | Strategic and operational managers  Staff  Researchers  Patient partners  Decision-makers |
| A willingness to innovate and support from decision-makers | - Target our need as a ministry and support the project financially and wait for the results of the project to see how we can use what has been developed in this project. (a representative of the decision-makers)  - When you do a transformation project you have to have the capacity to carry it through all the stages. And often I see that people talk a lot about projects but don't have the capacity to carry them through to completion. (a manager) | Decision-makers  Strategic managers |
| A private partner able to support technological innovation | - Having a private company capable of providing technological solutions. So that's the interest that we saw and the learning that we could do as an office afterwards. (a representative of the decision-makers, 27DM)  - Artificial intelligence is still very vague. But I am sure that what we can do with it can only be positive. (a physician, 02MD)  - For me, technology has to be used. And it's like technology can be a super important input. In addition to that, we bring value to the trades, to the professions and often people when they ask questions about their work... we can spend more time with people. We bring value to the job; we revalue the job. (a patient partner, 23PA) | Decision-makers  Staff  Private partner  Patient partners |
| **The implementation process enablers** | |  |
| A mobilising shared objective | - We are going to have to bring about change, focus it a lot for the patient if we bring the patient into our discussions. (a strategic manager, 34SM)  - The patient should really be at the heart of everything. Stop making people come to the emergency room ten times because it's the only option they have. Stop making them walk back and forth because they have to have this and that test. They have to repeat the same information every time they meet a new professional. (a researcher, 25RS)  - I think that somewhere along the line, when you create an interesting environment for everybody for the benefit of the patient. It's that person who's going to and the carers who are going to benefit in the end. (a patient partner, 22PA)  - The objective must be to help patients. In my opinion, a project like this, the centre of the project, the heart of the project, is the patient. (a physician, 24MD) | Strategic managers  Researchers  Staff  Patient partners |
| The involvement of patient partners | - I think that the secret, which was not present in the other projects, is precisely the fact of having patient partners, who will be able to, who will succeed in making these people talk. And we have to work together, we have to share our data. And maybe this tool will become the tool that will be shared by all. But that's my dream. (a researcher, 25RS)  - I think we need to promote patient partners as well, this is another spearhead that is super important in the ecosystem, in everything that is a partner we should prioritise it. (a patient partner, 23PA) | Decision-makers  Researchers  Patient partner |
| Strong leadership at all levels | - It's precisely the fact that it's someone like X who is connected, if you like, who will be able to, if anyone can succeed, it's him. (a researcher, 25RS)  - It takes good leadership and commitment from everyone. Good leadership promotes the commitment of the resources involved. Transparency, ethics as we are going to use technology. (a patient partner, 22PA) | Researchers  Strategic managers  Patient partners |
| Capitalise on the motivations and solutions of the staff | - For me, the manager, it will be extremely difficult. I think that my vision would be to bring this with them so that we can really co-construct it with the teams. That's really important so that we can get support, that's for sure. (an operational manager, 01OM)  - For the day and evening shifts, it's certain that finding a way of reducing the time spent on the file would be more effective because we could spend more time with the patient and take more care of him. They could make him walk more, stimulate him a bit more so that he decompensates a bit less. (a caregiver, 10CG)  - Involvement in the proposal, in the validation of the feedback, to have key people and to involve them because I believe that it is going to be a great risk to come up with a model which, yes, perhaps, responds to the best practices, the data in the literature and mathematically we are proving things, but in order for it to be applicable in the field, it is going to be necessary for the teams in the field to be well involved in the validation of possible solutions. (an operational manager, 29OM)  - Decrease the clerical work also of nurses and nursing assistants. I have the impression that they have a lot of clerical work and little time at the bedside, so doing simple things, they have too many tasks too. (a physician, 38MD)  - And sometimes I don't even see the file, the day or so, because there's always someone who has it, but me, let's say, in the care. It's not optimal because my patient, I haven't even been able to read the doctor's note, I haven't been able to read where we're at, it's obvious that if there were some way of finding the information. (a caregiver, 12CG) | Strategic and operational managers  Staff |
| The commitment of fields champions for change | - I have X and Y who are here now because they will certainly be mobilising agents for the new practices... The key stakeholders in our success. I have my assistants who are day and night, these people. It's going to be a matter of keeping them with us as mobilising agents, well-informed about our project, because these people are on the ground and they're going to be able to help us in this process too. (a operational manager, 01OM) | Strategic and operational managers |
| A capacity for continuous adaptation of the project | - So, I think it's also important to be able to have moments of introspection where you take a step back and say, "Are we getting the results we want? Are we going in the right direction, do we need to readjust? I think this is also important. (a funder's representative, 36FR) | Researchers  Decision-makers |
| **The challenges of implementation** | |  |
| Communication at all levels | - And often, if we can have actors from the health sector who may be off-putting in relation to research, for several, several reasons, but I think that we must remain really concrete with them. (a researcher, 30RS)  - I think that we have the challenge of saying that we have to be able to summarise the project in 30 seconds, so that it's clear to anyone. I think we have the challenge of saying that we must be able to summarise the project in 30 seconds, and then make it clear to anyone. (an operational manager, 29OM)  - It's important that everyone looks in the same direction and has similar working methods, that we are able to readjust together too, we mustn't each speak on our own. There are many communication issues, as I said. (a funder's representative, 36FR) | Researchers  Strategic and operational managers  Decision-makers |
| Availability of actors | - Then with the operational managers, they have to really work as a team to really move the project forward with the other unit managers who are concerned and the others. There has to be the time, the space and the capacity to really support the teams in the change. (a strategic manager, 37SM)  - You know, there are all sorts of things that can be done. But you know, nurses working the way they work, working overtime, they will never adhere to a continuing education program, like what they want to impose on them to arrive one, two or 3 hours before. Our challenge is to find solutions that can be seen in the workplace hours. But you can imagine that this is not easy. (a caregiver, 08CG) | Decision-makers  Strategic managers  Staff |
| Pace of implementation process | - We must not forget that the health sector is fast-paced and we like to act and we like to get results. Our patients arrive here and six days later they leave. So, for us, it's important to have quick results. (a strategic manager 32SM)  - In other non-health areas, there is less compliance, there is less legislation, there is less if-then, there are fewer constraints, which probably favours the completion of the work in a shorter time. (a representative of the private partner, 28PP) | Strategic managers  Private partner |
| Demonstrate value to all stakeholders | - It's just that if it doesn't add value to their own evaluation for their own... Or if it doesn't save them time directly, or you know, I'm not sure that at all levels, the patient benefit is going to outweigh the personal benefit. (a caregiver, 08CG)  - It's an optimised model, but I think it's going to be a matter of bringing it to the teams so that they feel that it's not a loss, but that there are gains as well, so that it's really something that we're going to have the team's support. (an operational manager, 01OM)  - People or actors in the health network are not at their first research project or transformation project or innovation project. And many projects were carried out with unquantified opportunities or those that were quantified did not quantify the effort required. So, in the end, people felt that they had invested a lot to get so little. (a strategic manager, 26SM) | Strategic and operational managers  Staff |
| Sustaining the project beyond the first results | - I think it's not just saying that and then walking away for years but it's good to measure satisfaction, the expected results. Follow-up at the end of the project. (an operational manager, 29OM)  - Unfortunately, it hasn't gone any further than that. If we bring in a new project. Everyone, I'm in the Researchgate (publication site), that's all very well, but that's where it stops and it has to continue. (a patient partner, 23PA) | Strategic and operational managers  Patient partners |
| **Barriers to implementation** | |  |
| Structural rigidity of professional practices | - And I've already seen that, trying to decompartmentalise the work of the inhaler and the work of the nurse, the only thing it does is create tension in the team. I don't think we should push this aspect too much. (a physician, 24MD)  - We talk about cross-curricular practices and modifying them, but I think we have to be careful about how... it can create really strong tensions if we want to propose important modifications that take away tasks from one and add them to others. (an operational manager, 29OM)  - Then there are also more administrative barriers because, depending on the proposed changes, there are collective agreements in place, there are unions that are also present, there are work organisations. I think that these will also be challenges, depending on what is proposed to change. (funder's representative, 36FR) | Operational managers  Staff  Decision-makers |
| Instability of human resources | - The fact of having instability in the team, you know, you know, field of practitioners, because it's often a rotation, it could be in the health sector, it could be the same, a rotation of nurses, a rotation of attendants, as you never have the same interlocutors, well there, at some point, it's an eternal restart. (an operational manager, 03OM) | Decision-makers  Operational managers |
| Low level of computerisation | - Basically, it was a project that is a little bit like putting technology at the service of the health system to free up nurses a little bit. But the results are far from what we could have done. (a researcher, 25RS)  - We talked about computerising certain processes, interoperating systems, that's a dream, because it's so outdated, the faxes, the laborious communication, the paper file that we look for all the time and can't find, and then finally we get the prescription an hour later. All this waste of time, we could do away with a lot of things at this level, it would be great. (an operational manager, 01OM)  - The prescription, one, an electronic prescriber. So, all the things that exist elsewhere in the world, which we haven't reached yet. So, when I arrive in the file click click, so that, then it minimizes the errors also. My writing is not always beautiful, etc. It minimises. So, I think these are things that should be improved. (a physician, 38MD) | Researchers  Operational managers  Staff |
| **The perspective of the replication and scaling up** | |  |
| A desired and foreseen replication | - In fact, these are good projects, but that's where it ends. It's deployment. That's where the frustration comes from. (a patient partner, 23PA) | Decision-makers  Patient partners  Private partner |
| Development of the methodological skills | - Positive outcomes for me, no matter what happen until the end, I feel that I will learn a lot during the whole process. (a researcher, 30RS)  - To meet often between researchers, you know, to try to talk as simply as possible about our difficulties, about what was happening, to realise that there were several shared things and that when we think together we can have a rapid solution that benefits everyone instead of everyone staying in their own corner and then trying to put things together. (a researcher, 31RS) | Decision-makers  Researchers |
| Building on learning | - To take this knowledge, to transform it into practice, to convince our people to reflect on their own practices, to get them to work, in short to change their routine of action, their modus operandi and their routine of interaction, is a nasty challenge. Then replicate it on other pathways, disseminate the knowledge and scale it up. That's a big challenge. (a researcher, 35RS)  - So, we can really use the knowledge that is produced and perhaps share it with other specialised institutes and centres. So, we will make sure that we work on this with you. (a representative of the decision-makers, 27DM) | Researchers  Decision-makers  Private partner |
| Fear of knowledge loss | - Too often, we do things in health and then leave them in health. Then, with abbreviations that nobody understands, we don't use the right literacy on this health issue. They say they are doing it for the citizens, but it's not true. At the end of the day, we don't understand. (a patient partner, 23PA) | Decision-makers Patient partners |

Quotes translated from French. The quotes are from focus group and individual interviews and logbooks, different project phases and different individuals, but for confidentiality reasons specified only by staff category, manager, or member of the project’s core group.
